# Letter to the Editor, Regarding “Comparative Efficacy and Safety of Different Tenecteplase Doses With Alteplase in Acute Ischemic Stroke: A Systematic Review With Pairwise and Network Meta‐Analysis to Determine the Optimal Dose” Recently Published by Waseem and Colleagues

**DOI:** 10.1002/brb3.71055

**Published:** 2025-11-21

**Authors:** Senta Frol, Matija Zupan, Panagiotis Papanagiotou

**Affiliations:** ^1^ Dept of Vascular Neurology University Medical Center Ljubljana Ljubljana Slovenia; ^2^ Faculty of Medicine University of Ljubljana Ljubljana Slovenia; ^3^ Clinic for Diagnostic and Interventional Neuroradiology Klinikum Bremen Mitte Bremen Germany; ^4^ Department of Radiology Aretaieion University Hospital National and Kapodistrian University of Athens Athens Greece

**Keywords:** intraarterial, tenecteplase

Dear Editor,

We read with great interest the recent article by Waseem et al. (2025). This comprehensive systematic review and network meta‐analysis is, to our knowledge, the first to rigorously evaluate the dose‐specific efficacy and safety of intravenous (IV) tenecteplase (TNK) in comparison with alteplase (rt‐PA). We commend the authors for this important contribution, which provides timely guidance for clinicians as TNK increasingly emerges as the thrombolytic agent of choice in acute ischemic stroke (AIS). The findings of Waseem et al. (2025) highlight the superiority of TNK 0.25 mg/kg in achieving excellent functional outcomes, while reaffirming a comparable safety profile relative to rt‐PA. This is a crucial step forward in evidence‐based decision‐making and in standardizing thrombolytic practice globally.

While the review focuses on IV administration, it is increasingly important to consider the intra‐arterial (IA) use of TNK after or alongside thrombectomy, a topic of growing relevance in interventional practice. From an interventional neuroradiology perspective, TNK's higher fibrin specificity, single‐bolus kinetics, and prolonged half‐life make it particularly suitable for targeted IA application. Several clinical trials are currently underway investigating adjunctive IA TNK in patients with AIS undergoing thrombectomy (Frol et al. 2025), with ANGEL‐TNK (NCT05624190) already completed and published (Miao et al. 2025), the pilot BRETIS‐TNK study (NCT04202458) also completed (Zhao et al. 2023), BRETIS‐TNK II (NCT05657444) having finished enrollment with results pending, while the remaining trials—TECNO (NCT05499832), INSIST‐IT (NCT05657457), ATTENTION‐IA (NCT05684172), INSIST‐TNK (NCT04201964), RESCUE‐TNK (NCT05657470), and EXTEND‐AGNES TNK (NCT05892510)—are still ongoing without interim results available. The ANGEL‐TNK trial showed that IA TNK after successful recanalization improved microvascular reperfusion and outcomes without safety concerns (Miao et al. 2025), while BRETIS‐TNK suggested higher first‐pass reperfusion without increased symptomatic hemorrhage (Zhao et al. 2023). These findings reinforce the concept that pharmacologic augmentation with IA‐TNK may improve distal and microvascular reperfusion beyond the limits of mechanical thrombectomy (MT), particularly in cases of incomplete or slow‐flow reperfusion (mTICI 2b–2c).

Current clinical protocols illustrate marked heterogeneity in IA‐TNK dosing (Frol et al. 2025). The ALLY trial (NCT05172934) applies 1.5 mg boluses (up to 4.5 mg), while BRETIS‐TNK II (NCT05657444) uses a 4 mg bolus distal to the clot, followed by a short infusion. Infusion regimens vary, with INSIST‐IT and RESCUE‐TNK delivering 0.2–0.3 mg/min for 20–30 min and INSIST‐TNK 0.2–0.4 mg/min over 30–40 min. Weight‐based strategies include ATTENTION‐IA (0.0625 mg/kg, max 6.25 mg), ANGEL‐TNK (0.125 mg/kg, max 12.5 mg), and EXTEND‐AGNES TNK (0.062 mg/kg, max 6.25 mg). Finally, TECNO specifies microcatheter administration near the residual occlusion but does not predefine the dose. Some protocols adjust doses when IV was already administered or when treating posterior circulation strokes, typically favoring lower amounts. Taken together, these heterogeneous regimens underscore the current uncertainty surrounding the optimal IA TNK strategy (Frol et al. 2025). Although preliminary experiences suggest a favorable safety profile, only the results of ongoing trials will establish which dosing and delivery protocols provide the most effective and safe approach for clinical implementation. The considerable variability in trial designs and administration methods highlights the need for definitive evidence, and their forthcoming results are eagerly anticipated to guide optimal dosing strategies and clarify the role of IA‐TNK in the management of AIS.

This question on IA TNK doses is particularly pertinent in the era of neutral distal and medium‐vessel occlusion thrombectomy trials (Goyal et al. 2025; Psychogios et al. 2025; Clarençon 2024). As these studies suggest limitations of current mechanical strategies in smaller vessels, pharmacological augmentation with IA TNK could represent an important complementary pathway to improving outcomes.

We believe the next critical step is the systematic collection of real‐world data on IA‐TNK use. Fundamental questions regarding optimal dosing regimens, timing of administration, and patient selection remain unresolved. Large multicenter registries could generate essential preliminary evidence, which would not only inform clinical practice but also provide a robust foundation for the design of future prospective randomized trials.

Standardized registries integrating procedural and imaging endpoints from interventional centers will be essential to define the optimal role of IA‐TNK in endovascular stroke therapy. In conclusion, we congratulate Waseem and colleagues (Waseem et al. 2025) for their timely and clinically relevant review. Beyond establishing the role of TNK as a superior IV agent, we encourage the stroke community to also investigate and report on IA use. This could prove especially important after incomplete reperfusion in large vessel occlusions and in scenarios where MT alone fails to provide durable benefit.

## Author Contributions

Conceptualization: Senta Frol. Drafting of the manuscript: Senta Frol, Matija Zupan. Review and editing of the manuscript: Senta Frol, Matija Zupan, and Panagiotis Papanagiotou. Project supervision: Panagiotis Papanagiotou. Guarantor of the study: Senta Frol.

## Funding

The authors have nothing to report.

## Ethics Statement

The present research complies with the guidelines for human studies, and the research was conducted ethically in accordance with the World Medical Association Declaration of Helsinki.

## Consent

The authors have nothing to report.

## Conflicts of Interest

Senta Frol received speakers honoraria from AstraZeneca, Bayer, Boehringer Ingelheim. Matija Zupan received speakers’ honoraria from AstraZeneca, Bayer, Boehringer Ingelheim. Panagiotis Papanagiotou received speakers’ honoraria from AstraZeneca, Bayer, Boehringer Ingelheim.

## Data Availability

The authors have nothing to report.
